# Disseminated Enterovirus Infection in a Patient Affected by Follicular Lymphoma Treated with Obinutuzumab: A Case Report and a Narrative Review of the Literature

**DOI:** 10.3390/medicina60030495

**Published:** 2024-03-18

**Authors:** Tommaso Lupia, Silvia Corcione, Elena Staffilano, Roberta Bosio, Antonio Curtoni, Alessandro Busca, Francesco Giuseppe De Rosa

**Affiliations:** 1 Unit of Infectious Diseases, AOU City of Health and Sciences, 10100 Turin, Italy; silvia.corcione@unito.it (S.C.); francescogiuseppe.derosa@unito.it (F.G.D.R.); 2 Department of Medical Sciences, Infectious Diseases, University of Turin, 10126 Turin, Italy; elena.staffilano@unito.it (E.S.); roberta.bosio@unito.it (R.B.); 3 Microbiology and Virology Unit, AOU City of Health and Sciences, 10100 Turin, Italy; antonio.curtoni@unito.it; 4 Department of Oncology, Stem Cell Transplant Center, Città Della Salute e Della Scienza Hospital, 10100 Turin, Italy; abusca@cittadellasalute.to.it

**Keywords:** enterovirus, obinutuzumab, lymphoma, encephalitis, hematological malignancy

## Abstract

*Background and Objectives*: the principal purpose of this literature review is to cluster adults with hematological malignancies after treatment or on maintenance with obinutuzumab who experienced disseminated EV infection to understand clinical characteristics and outcome of this rare condition in these patients. We report the first clinical case of a male affected by follicular lymphoma treated with immune-chemotherapy including obinutuzumab who was affected by disseminated EV infection with cardiovascular involvement. *Materials and Methods*: this narrative review summarizes all the research about disseminated EV infection in immunosuppressed adult patients treated with obinutuzumab from January 2000 to January 2024 using the Scale for the Assessment of Narrative Review Articles (SANRA) flow-chart. We performed a descriptive statistic using the standard statistical measures for quantitative data. *Results*: we included six studies, five case reports, and one case report with literature analysis. We collected a total of seven patients, all female, with disseminated EV infection. The most common signs and clinical presentations of EV infection were fever and encephalitis symptoms (N = 6, 85.7%), followed by hepatitis/acute liver failure (N = 5, 71.4%). *Conclusions*: onco-hematological patients who receive immune-chemotherapy with a combination of treatments which depress adaptative immunity, which includes the antiCD20 obinutuzumab, could be at higher risk of disseminated EV infection, including CNS and cardiac involvement.

## 1. Introduction

The term enterovirus (EV) was introduced in 1957 to unify in one family the polioviruses, Coxsackie A and B, and echoviruses [1]. Nowadays the EV is a genus characterized by 13 species, seven causing human diseases, Enterovirus A–D and Rhinovirus A–C, belonging to the *Picornaviridae* family [2].

These viruses replicate in the oropharynx and gastrointestinal (GI) tract and are normally spread by fecal-hand-oral contamination [2]. The viruses are frequently widely disseminated in the summer and fall during the year, circulating in particular among young children, causing both apparent and inapparent infection [3]. They can cause nonspecific febrile diseases, sometimes with rash, aseptic meningitis, paralytic disease, pericarditis, and myocarditis, as well as some specific syndromes such as herpangina and hand-foot-and-mouth disease [2,3]. There is an overlap in biologic behavior, and the same syndrome can be caused by many different agents [2]. Antibodies are the main form of defense against EVs, and severe, chronic, and disseminated enteroviral infections are generally limited to neonates or patients with profound B-cell deficiencies [3]. Within the B-cell deficiencies, we should mention follicular lymphoma (FL) which is a systemic neoplasm of the lymphoid tissue and represents around 5% of all hematological neoplasms and around 20–25% of all new non-Hodgkin lymphoma diagnoses in Western countries [4], consequently it means that FL is the most common subtype of indolent non-Hodgkin lymphomas. The treatment of FL normally consists of immune-chemotherapy and often brings to long-lasting remissions [5]. Although rituximab-based immunochemotherapy has improved outcomes in patients with follicular lymphoma [6], progression-free survival with glycoengineered type II anti-CD20 monoclonal antibody obinutuzumab was shown to be better, in combination with either bendamustin e or anthracycline-based chemotherapy [5]. The treatment with obinutuzumab is generally well tolerated, even if the adverse events are more common that with rituximab regimens [6]. Here, we present the case of a 34-old man with FL treated with obinutuzumab that develops a disseminated EV infection with pericarditis.

## 2. Materials and Methods

**Figure 1:**
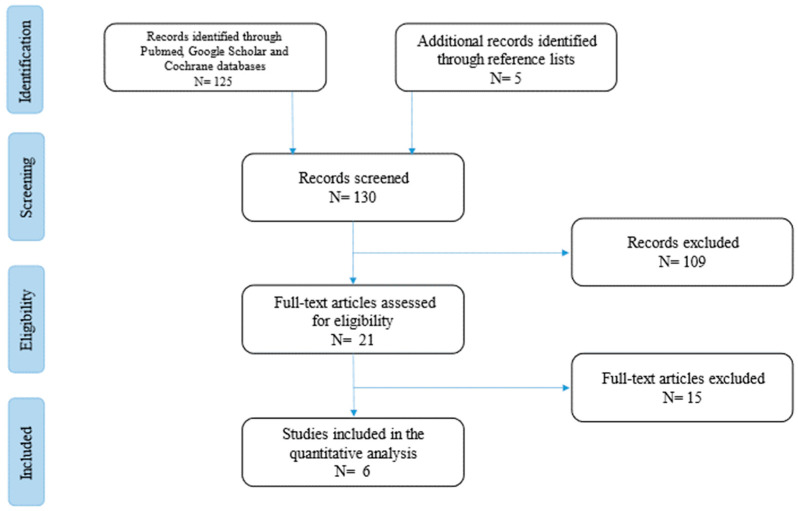
Flow-chart of the studies revised in the narrative review.

The current narrative review followed the Scale for the Assessment of Narrative Review Articles (SANRA) flow-chart (Figure 1) [7].

The main aim of this work was to summarize current evidence on EV infections in patients treated with obinutuzumab in immunosuppressed adult patients to understand clinical characteristics, treatments, and outcomes.

A search was run on Cochrane, PubMed, and Google Scholar using the terms (‘Enterovirus’ [Mesh]) AND (‘Obinutuzumab’ [Mesh]) OR (‘Enterovirus’ [Mesh]) AND (‘CD20′ [Mesh]), in English. Results were limited to those published between 1 January 2000 and 1 January 2024. Studies were filtered for practice guidelines, guidelines, meta-analyses, systematic reviews, narrative reviews, case series, and case reports. Therefore, we have filtered results to include only human and adult patients.

Our search strategy permitted the identification of 130 papers, of which 124 were excluded by title and abstract evaluation. Then, the reviewers studied titles and abstracts. Subsequently, 6 papers were included. Finally, quality assessment of full-text studies was performed by two independent reviewers (ES and TL). Researchers reviewed the summary of all articles sought and ultimately used data from full articles to compile this review paper. Researchers assessed the inclusion of all titles and abstracts without language limitations in English. We duplicated other studies previously included and excluded papers with no methods described, along with papers not strictly related to the aim of the study and according to journal importance and the number of references.

We performed descriptive statistics on the entire study population. Data were analyzed using standard statistical methods. Variables were described with medians, absolute values, and rates.

## 3. Case Report

In October 2022, a 34-year-old man requested medical attention because of a headache, fever, and asthenia lasting five days. His previous clinical history was remarkable for the diagnosis, on 20 February 2022, of FL grade 2 according to 5th WHO classification of hematolymphoid tumors [8], treated with induction immunochemotherapy and obinutuzumab; he was in complete remission of the disease, and a maintenance program with obinutuzumab was planned. He was on therapy with isoniazid for latent tuberculosis. He had two episodes of diarrhea in the previous days and reported similar symptoms in one of his two young children. At first assessment, he presented as vigilant, oriented, tachycardic, and febrile, and he complained of diffused abdominal pain on deep palpation.

He underwent two sets of blood cultures, negative for bacteria and fungi; in the suspect case of CNS involvement, a brain computed tomography (CT) was requested, which was negative for new abnormalities. The jolt sign was positive, a lumbar puncture was done, and the cerebrospinal fluid (CSF) was clear. It had 31 cells/mm^3^ of mononuclear cells, 88 mg/mL of proteins, and 69 mg/dL of glucose. On CSF, reverse transcriptase (RT) PCR for West Nile virus, Usutu virus, were negative, while identified EV. Quantitative Enterovirus ELITe MGB RT-PCR (ElitechGroup, Turin, Italy) confirmed multiplex results and detected 649 copies/mL of RNA on CSF, which led to the diagnosis of EV meningoencephalitis. Coproculture and stool ova and parasite tests were negative. He was treated with supportive treatment, plus cotrimoxazole and acyclovir for prophylaxis. Since the persistence of the fever, he was subjected to further investigation: total-body CT, brain magnetic resonance imaging (MRI), and echocardiography were unremarkable. After four days, he triggered large-QRS tachycardia with a new-onset complete right bundle branch block and a heart rate of 110 beats per minute. The echocardiography was done again, and this time it showed that the ejection fraction was 35% lower, and the septal, inferior, and lateral heart areas were not contracting properly. There was also an increase in the myonecrosis enzymes (isoenzyme MB of the creatine-phosphokinase 98.85 ng/mL; high-sensitive troponin 19,073 pg/m). The day after, he showed the first signs of heart failure with hypotension and an EF of 25–30% on the echocardiogram and required admission to the Intensive Care Unit (ICU) for cardiogenic shock. Immediately after, the patient went into cardiovascular arrest, requiring orotracheal intubation and extracorporeal circulation. He was treated with intravenous immunoglobulin (IVIg), although his serum IgG levels were within normal range (8.05 g/L), and high dose of intravenous corticosteroids, with subsequent progressive improvement. He was discharged from the ICU after two weeks with a marked diffused hypokinesia of the left chambers and an EF of 40%.

In conclusion, he had a disseminated EV infection complicated with acute infective myopericarditis. The diagnosis was subsequently confirmed by heart MRI.

**Figure 2:**
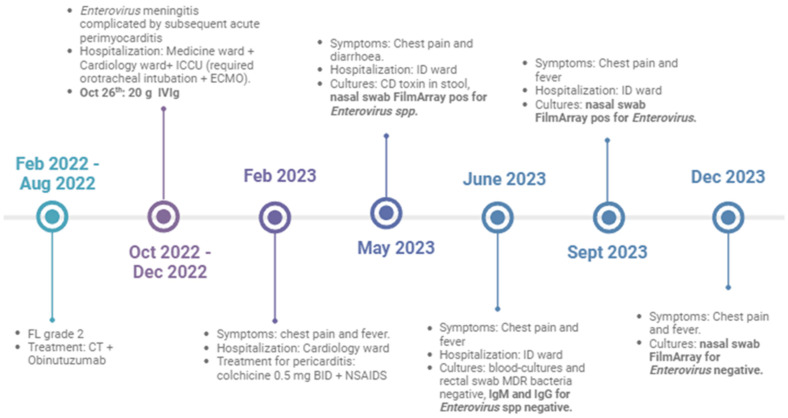
Timeline of Clinical and Microbiological Presentation of Disseminated Enterovirus infection after Obinutuzumab. Abbreviation: FL: follicular lymphoma; CT: chemotherapy; ICCU: intensive coronary care unit: ECMO: extra-corporeal membrane oxygenation; IV: intravenous; IVIg: intravenous immunoglobulin; BID: bis in die: NSAIDs: non-steroidal anti-inflammatory drugs; ID: Infectious Disease; CD: Clostridioides difficile; MDR: multi-drug resistant. Created with BioRender.com (accessed on 17 March 2024).

Before being discharged from the hospital, he required a period of motor rehabilitation for the onset of sensory-motor axonal polyneuropathy. He was discharged with a 2-month cardiological follow-up visit and echocardiogram. Subsequently, he experienced frequent relapses with thoracic pain accentuated by supine position and breathing associated with fever and inflammatory indices rising. He recovered in the cardiology ward and was discharged with a diagnosis of pericarditis and an indication for therapy with colchicine 0.5 mg BID plus a nonsteroidal anti-inflammatory drug (NSAID). The patient later came to our attention for infectious complications such as sepsis caused by multi-resistant bacteria and an episode of Clostridioides difficile infection (CDI). EV was isolated in the nasopharynx swab various times (Figure 2), and he has repeated cardiologic assessments, which indicated pericarditis with an EF of about 40%. He was subjected to positron emission tomography (PET-TC), which confirmed blurred accumulation of the fluorodeoxyglucose F-18 in the mediastinum at the level of the pericardium. In the end, he was diagnosed with chronic EV pericarditis and treated with colchicine, NSAIDs, and a high dosage of corticosteroids (he started with 25 mg of 1 pill TID for 10 days following low tapering). Remarkably, when we tested quantitative EV IgM and IgG on serum, he has not developed any specific antibody response to EV and it remains detectable in the airways for approximately one year; molecular panel for high-airways pathogens (FilmArray^®^ Respiratory Panel, bioMérieux, Marcy l’Etoile, France) on nasal swab resulted negative for EV on December 2023. He was referred to the Reference Centre for Pericarditis of Turin to be a candidate for anti-interleukin 1 treatment in the major hypothesis of refractory pericarditis once he discontinued corticosteroids; at the moment, this was not possible for the relapses of the pericarditis symptoms when he tried to reduce corticosteroids dosage.

## 4. Results

We have included six studies in the final analysis of this review, five case reports, and one case report with a literature review. From the studies included, we collected seven patients reporting obinutuzumab-related infections due to EVs. All the patients were female (N = 7, 100%), with a median age of 45 years (range 33–67), as reported in Table 1.

All the patients included reported an active hematological malignancy in their medical history (Table 1) and the most frequent diagnosis was non-Hodgkin lymphoma, notably FL (N = 5, 71.4%). No data on other concurrent comorbidities was reported in the cases reviewed. 

Moreover, obinutuzumab was prescribed in both therapeutic and maintenance regimens, as reported in Table 1, despite the fact that the obinutuzumab dosage was shown only by the Heger et al. group (i.e., 1000 mg every 8 weeks). Recent previous treatments reported before obinutuzumab were bendamustine (N = 5, 71.4%), venetoclax (N = 1, 14.3%), CHOP (N = 1, 14.3%), and rituximab (N = 1, 14.3%) alone or in combination therapy (Table 1). EV infections occurred with a median time after the last dose of obinutuzumab of 48 weeks (36–48 weeks), despite the fact that in two cases, the exact median time between infection and anti-CD20 therapy was not reported (Table 1). The most common signs and clinical presentations of EV infection were fever and encephalitis symptoms (N = 6, 85.7%), followed by hepatitis/acute liver failure (N = 5, 71.4%). The diagnosis of EV infection was performed in all the cases reported with molecular PCR on different body fluids. EV RNA in CSF and blood resulted positive in 85.7% and 71.4% of the cases, respectively. Moreover, different papers reported IgM, IgG, and IgA low levels to confirm hypogammaglobulinemia after obinutuzumab in five patients; two patients reported both low IgM and IgG levels. None of the patients were treated with specific anti-EV antivirals, despite the fact that all the patients underwent a five-day course of EV Ig. The mortality rate in the cohort of patients was 14.3% (N = 1), and this death was directly connected to the EV infection. No chronic EV infections were reported barring the experience of McNeill et al. [8] in which EV was detected 1 month after the acute presentation of disseminated disease.

One of the worst consequences of EV infections is encephalitis that can lead to severe neurological sequelae. The infection of brain and of Central Nervous System (CNS) cells induces cytokines and growth factors secretion and the recruitment of immune cells. The inflammatory response in the CNS can lead to neurological impairment such as speech disorders, memory loss, motor dysfunction, but also depression and Alzheimer’s disease.

## 5. Discussion

Disseminated EV infections in the immunocompromised population occurring after obinutuzumab treatment are a rare, fast-growing infectious complication with a related high comorbidity and mortality. In this paper, we have reported a recent case of EV disseminated disease after obinutuzumab and a comparative review of the cases available in the literature until January 2024.

Most of the cases collected were published recently, between May 2014 and November 2023 [9,10,11,12,13,14]. The Food and Drug Administration (FDA) and the European Medicine Agency (EMA) had approved obinutuzumab for CLL in November 2013 and July 2014, respectively [15,16]. Over the following years, treatment indications for obinutuzumab have been expanded, and its use has been more frequent in lymphoma malignancies [6,17].

Among the seven cases of disseminated EV infections described to date in the literature, we have found a total of cases in the female sex. These data are in contrast with previous reports regarding sex prevalence among EV infected patients. In fact, according to a wide surveillance report from the United States, among 52,812 EV detections between 1970 and 2005, females accounted only for 43.0% of reports with known sex [18]. Moreover, males are more likely to have severe diseases after EV infection than females [19]. In our case report, we have reported at our knowledge, the first disseminated EV disease in a male patient after obinutuzumab infection.

Furthermore, the age of our patient is in line with the reviewed papers [9,10,11,12,13,14]. Despite that, according to Khetsuriani et al., EV infections occurred notably in pediatric patients; in fact, only 4349 (17.3%) of cases were detected in persons aged >20 years [18]. According to a previous review by Grisariu and colleagues [20], in which the authors collected six cases of EV infections during rituximab treatment in NHL and reviewed seven cases from the literature (i.e., for a total of thirteen cases), it can be noticed that our cohort has a higher median age (62 vs. 45 years) and a wider range of ages (42–80 vs. 33–67 years).

In our series, most patients were treated with obinutuzumab for NHL (85.7%), especially follicular lymphoma (71.4%), which is also the diagnosis of our patient. On the contrary, in Grisariu and colleagues review, all the hematological malignancies were NHLs, and follicular lymphoma accounted only for 46.1% of cases presented (6/13) [20].

Furthermore, similarly to our review, the cases presented by Grisariu et al. [20] did not report past medical history other than hematological diseases. The lack of data on comorbidities in these patients does not allow complete risk stratification of this population. In fact, other comorbidities or immunosuppression states could increase the risk of EV infection. In our case report, we have collected past medical history of the patient that was unremarkable, despite those patients with congenital isolated B-cell deficiency or combined immunodeficiencies, HIV patients, and solid organ recipients are at considerable risk of EV infection [21], such as diabetes mellitus or autoimmune diseases [22]. 

In our series, most of the patients underwent previous CHT, notably with bendamustine, before obinutuzumab. Bendamustine was administered also in our patient before obinutuzumab maintenance. Furthermore, bendamustine had a complex immunosuppression profile and infection risk for the patient, such as myelosuppression, including lymphopenia and a low CD4+ T count, and hypogammaglobulinemia [23]. It might reflect the importance of humoral immune response in EV infections, due to the fact that most of EV disseminated infections had occurred when both T helper CD4+ lineage and B-cells response are depressed. To our knowledge, no cases of EV infections were reported with bendamustine therapy alone. Interestingly, no patients presented by Grisariu et al. in their report underwent bendamustine treatment [20].

Among the published reports regarding hematological patients treated with obinutuzumab, the minimum and maximum time from treatment to symptoms were 36 and 68 weeks, respectively. In our case report, EV disseminated disease has been developed earlier, only 28 weeks after the start of obinutuzumab. In the Grisariu et al. review, the maximum time for presentation was 24 weeks [20]. Amitai et al. recently published a meta-analysis regarding the safety profile of obinutuzumab: in most of studies included this monoclonal antibody was compared with rituximab [24]. The authors estimate a favored increase in the grade 3–4 infection rate with obinutuzumab, although this was not statistically significant (RR 1.17 [95% CI, 1.0–1.36], 4 trials, 3429 patients) [24]

Regarding clinical presentations we have found fever, encephalitis (85.7%; N = 6) and hepatitis/acute liver failure (71.4%; N = 5) as the most common signs and symptoms of EV infections. Similar findings and prevalence, especially for fever and neurological involvement, were reported in the review signed by Grisariu and colleagues [20]. Although EVs infection, in particular coxsackievirus B (CVB), are linked to the development of myocarditis, cardiovascular presentation with pericarditis and/or myocarditis, as reported in our case report, was not presented previously in other case reviewed after anti-CD-20 therapies. 

Moreover, diagnosis was made in our cohort by EV RNA detected in CSF and blood in the 85.7% and 71.4%, respectively. Compared to Grisariu et al., in which EV RNA on CSF was performed and detectable in the whole hematological population (N = 13, 100%), plasma CSF data are lacking [20].

Hypogammaglobulinemia was reported frequently in our cohort of patients (N = 5, 71.4%), despite that data on immunoglobulin levels were rarely reported, similar to Grisariu et al. work (N = 11, 84.6%) [20]. Both primary and secondary hypogammaglobulinemia can increase the number of bacterial and viral infections. In a study conducted by Kainulainen et al., 12 adults with primary hypogammaglobulinemia were followed for 12 months and were tests with nasal swabs and sputum samples [25]. Respiratory tract viruses were found in sputum in 54% of the infections. The most common virus was Rhinovirus. In more than half of our patients, rhinoviral PCR results stayed positive for more than 2 months [25]. 

Although most enterovirus infections cause only mild and self-limiting diseases, the large number of cases and high prevalence of enterovirus infections throughout the world highlight the need for specific antiviral drugs against enteroviruses. Li et al. showed the possible role of molnupiravir in vitro and in vivo as a drug with an excellent broad-spectrum anti-enterovirus activity [26]. Epstein and colleagues showed a case report of an old woman treated with pocapavir and immunoglobulins as compassionate use [27]. Immunoglobulin therapy for EV infections was administered to the whole population of patients treated with obinutuzumab and no specific antiviral therapy was reported. In Grisariu et al. work, immunoglobulins were administered in 84.6%, furthermore acyclovir (N = 2), pleconaril (N = 2), and ganciclovir (N = 1) were reported in the treatment regimens of patients treated with rituximab [20]. Immunoglobulin treatment for EV sever infections seems to be used particularly among children. Amdani and his group used immunoglobulin and pocapavir to treat fulminant neonatal enteroviral myocarditis in monochorionic diamniotic twins with success [28].

In our case report, our patient presented clinical symptoms of chronic recurrent pericarditis for about eleven months after disseminated diseases: no similar chronic presentations were found in cases reviewed. Interestingly, in the Grisariu and colleagues’ paper, two patients reported EV-related post-acute symptoms up to 12-months after disseminated disease [20]. There is quite an old but interesting article published by the Lancet showing different IgM responses in chronic and acute pericarditis. In the study, EV-specific IgM responses were demonstrated in 9 of 14 (64%) patients with chronic relapsing pericarditis and this finding suggests persistent enterovirus infection. These IgM responses persisted for 1 to 10 years after the onset of symptoms. On the other hand, patients with acute enterovirus infections, including acute pericarditis, had transient responses [29].

We have found, fortunately, a low rate of mortality after EV disseminated disease; on the contrary, death related to EV infection was higher, around 46% [20].

## 6. Conclusions

In conclusion, disseminated EV infection seems to be a prerogative of patients affected by hematological malignancies, in the majority of cases NHL, especially FL, who are treated with regimens which deeply depress adaptive immunity on different targets leading to a long-lasting and deeply immunocompromised status, which includes obinutuzumab. This is also reflected by the timing of disseminated EV infection presentation, which can occur several weeks after obinutuzumab starts.

Disseminated EV infection is prevalent in the female sex; nevertheless, EV infection is more frequent in males and with higher risk of severe progression.

The presented case is the first reported case of disseminated chronic EV infection in a males treated with obinutuzumab, who showed cardiac involvement with chronic pericarditis. Moreover, the symptoms onset was earlier than the others reported in literature.

At the moment, there is no specific treatment for EV infection; some antivirals show efficacy as intravenous immunoglobulins, although the evidence is scarce.

At last, all cases of disseminated EV infections were reported after obinutuzumab was commercialized and its treatment indications expanded, so patients who undergo combination immune-chemotherapy, including obinutuzumab, may be at higher risk to develop disseminated or chronic EV infection.

## Figures and Tables

**Table 1:** Clinical, microbiological, and treatment data collected from the studies reviewed [9].

Authors	Year	Type of Article	Number of Patients	Disease for Obinutuzumab	Obinutuzumab Indication	Dose	Recent Previous Treatment	Sex	Age	Comorbidities	Disease from Obinutuzumab Start	Clinical Presentation	Enterovirus Diagnosis	Hypogammaglobulinaemia	Treatment	Outcome	Enterovirus-Related Death	Chronic
Heger et al. [9]	2019	Case Report	1	NHL (FL)	M	1000 mg every 8 weeks	Rituxima, bendamustine	F	45	NR	48 weeks	Fever, acute liver failure, encephalitis and colitis	RNA on blood, CSF, stool	Yes (IgG)	Ig	Death	Yes	No
Dendle et al. [10]	2015	Case Report	2	NHL (FL)	T + M	NR	bendamustine	F	63	NR	44 weeks	Hepatitis, dermatomyositis	RNA on blood, Nasal swab, stool, muscle	No	Ig	Alive	No	No
				NHL (FL)	T + M	NR	bendamustine	F	35	NR	48 weeks	Fever, hepatitis and encephalitis	RNA on blood, CSF, stool	No	Ig	Alive	No	No
Eyckmans et al. [11]	2014	Case Report	1	NHL (N.S.)	T + M	NR	bendamustine	F	67	NR	7 doses (maintenance)	Fever and encephalitis	RNA on CSF	Yes (IgG)	Ig	Alive	No	No
Blau et al. [12]	2023	Case Report	1	CLL	T	NR	venetoclax	F	67	NR	36 weeks	Fever, acute liver failure, encephalitis, arthralgias and myalgias	RNA on CSF	Yes (IgG and IgM)	Ig	Alive	No	No
Wagner et al. [13]	2021	Case Report and Review	1	NHL (FL)	T	NR	CHOP	F	33	NR	68 weeks	Fever, skin lesions and encephalitis	RNA on blood, CSF, stool	Yes (IgG and IgM)	Ig	Alive	No	No
McNeil et al. [14]	2021	Case Report	1	NHL (FL)	T	NR	bendamustine	F	40	NR	5 doses (induction)	Fever, hepatitis and encephalitis	RNA on blood, CSF, stool	Yes (IgG)	Ig	Alive	No	Detected for 1 month

Abbreviations: FL: follicular lymphoma, CLL: chronic lymphocytic leukaemia; NHL: non-Hodgkin lymphoma; T: therapeutic; M: maintenance; NR: not reported; F: female; CHOP: Cyclophosphamide, hydroxydaunorubicin, vincristine, and prednisone; CSF: cerebrospinal fluid; Ig: immunoglobulin. N.S.: not specified.

## Data Availability

Data are available from the authors upon request.

## References

[1] Committee on the Enteroviruses, National Foundation for Infantile Paralysis (1957). The Enteroviruses. Am. J. Public Health Nations Health.

[2] Baggen J., Thibaut H.J., Strating J.R.P.M., van Kuppeveld F.J.M. (2018). The life cycle of non-polio enteroviruses and how to target it. Nat. Rev. Microbiol..

[3] Maheshwari A., Motta M., Singh S., Kasniya G., Mane S.S., Cartaya S., Rahman M.M., Dudeja P. (2022). Enteroviral Infections in Infants. Newborn.

[4] Jubelt B., Lipton H.L. (2014). Enterovirus/Picornavirus infections. Handbook of Clinical Neurology.

[5] Misbah S.A., Spickett G.P., Ryba P.C.J., Hockaday J.M., Kroll J.S., Sherwood C., Kurtz J.B., Moxon E.R., Chapel H.M. (1992). Chronic enteroviral meningoencephalitis in agammaglobulinemia: Case report and literature review. J. Clin. Immunol..

[6] Carbone A., Roulland S., Gloghini A., Younes A., von Keudell G., López-Guillermo A., Fitzgibbon J. (2019). Follicular lymphoma. Nat. Rev. Dis. Prim..

[7] Baethge C., Goldbeck-Wood S., Mertens S. (2019). SANRA—A scale for the quality assessment of narrative review articles. Res. Integr. Peer Rev..

[8] Li W., Li W. (2022). The 5th Edition of the World Health Organization Classification of Hematolymphoid Tumors. Leukemia.

[9] Heger J., Eichenauer D.A., Kasper P., Böll B., Shimabukuro-Vornhagen A., Kochanek M. (2019). Fatal disseminated enterovirus infection in a patient with follicular lymphoma undergoing obinutuzumab maintenance therapy. Eur. J. Haematol..

[10] Dendle C., Gilbertson M., Korman T.M., Golder V., Morand E., Opat S. (2015). Disseminated Enteroviral Infection Associated with Obinutuzumab. Emerg. Infect. Dis..

[11] Eyckmans T., Wollants E., Janssens A., Schoemans H., Lagrou K., Wauters J., Maertens J. (2014). Coxsackievirus A16 Encephalitis during Obinutuzumab Therapy, Belgium, 2013. Emerg. Infect. Dis..

[12] Blau B.J., Bass A.R. Chronic Enterovirus Infection Manifesting as Diffuse Myalgias and Arthralgias in a 67-Year-Old Woman Receiving Obinutuzumab. Grand Rounds from HSS: Management of Complex Cases, 2023, Volume 12, Issue 3. https://www.hss.edu/conditions_complexcase-chronic-enterovirus-infection.asp.

[13] Wagner J.N., Leibetseder A., Troescher A., Panholzer J., von Oertzen T.J. (2021). Characteristics and therapy of enteroviral encephalitis: Case report and systematic literature review. Int. J. Infect. Dis..

[14] McNeil T., Hannah R., Hui C., Qiao M. (2022). Disseminated enterovirus infection in a patient treated with obinutuzumab. J. Med. Virol..

[15] https://fda-approves-gazyva-chronic-lymphocytic-leukemia-3955.html.

[16] https://www.ema.europa.eu/en/medicines/human/orphan-designations/eu-3-12-1054.

[17] Marcus R., Davies A., Ando K., Klapper W., Opat S., Owen C., Phillips E., Sangha R., Schlag R., Seymour J.F. (2017). Obinutuzumab for the First-Line Treatment of Follicular Lymphoma. N. Engl. J. Med..

[18] Khetsuriani N., Lamonte-Fowlkes A., Oberst S., Pallansch M.A., Centers for Disease Control and Prevention (2002). Enterovirus Surveillance–United States, 1970–2005. Morbidity and Mortality Weekly Report. Surveillance Summaries.

[19] Nikonov O.S., Chernykh E.S., Garber M.B., Nikonova E.Y. (2017). Enteroviruses: Classification, diseases they cause, and approaches to development of antiviral drugs. Biochemistry.

[20] Grisariu S., Vaxman I., Gatt M., Elias S., Avni B., Arad A., Pasvolsky O., Raanani P., Paltiel O. (2017). Enteroviral infection in patients treated with rituximab for non-Hodgkin lymphoma: A case series and review of the literature. Hematol. Oncol..

[21] Dunn J.J. (2016). Enteroviruses and Parechoviruses. Microbiol. Spectr..

[22] Kennedy P.G.E. (2021). An overview of viral infections of the nervous system in the immunosuppressed. J. Neurol..

[23] Gafter-Gvili A., Polliack A. (2016). Bendamustine associated immune suppression and infections during therapy of hematological malignancies. Leuk. Lymphoma.

[24] Amitai I., Gafter-Gvili A., Shargian-Alon L., Raanani P., Gurion R. (2021). Obinutuzumab-related adverse events: A systematic review and meta-analysis. Hematol. Oncol..

[25] Kainulainen L., Vuorinen T., Rantakokko-Jalava K., Österback R., Ruuskanen O. (2010). Recurrent and persistent respiratory tract viral infections in patients with primary hypogammaglobulinemia. J. Allergy Clin. Immunol..

[26] Li Y., Liu M., Yan Y., Wang Z., Dai Q., Yang X., Guo X., Li W., Chen X., Cao R. (2022). Molnupiravir and Its Active Form, EIDD-1931, Show Potent Antiviral Activity against Enterovirus Infections In Vitro and In Vivo. Viruses.

[27] Epstein S., Thakkar R., Fong K.T., Ng J., Bearden D.R., Mishra N., Thakur K.T., Riley C.S. (2022). Compassionate-use pocapavir and immunoglobulin therapy for treatment of rituximab-associated enterovirus meningoencephalitis. J. Neurovirol..

[28] Amdani S.M., Kim H.S., Orvedahl A., John A.O., Said A., Simpson K. (2018). Successful treatment of fulminant neonatal enteroviral myocarditis in monochorionic diamniotic twins with cardiopulmonary support, intravenous immunoglobulin and pocapavir. BMJ Case Rep..

[29] Muir P., Tilzey A., English T., Nicholson F., Signy M., Banatvala J. (1989). Chronic relapsing pericarditis and dilated cardiomyopathy: Serological evidence of persistent enterovirus infection. Lancet.

